# A comparison of SNPs and microsatellites as linkage mapping markers: lessons from the zebra finch (*Taeniopygia guttata*)

**DOI:** 10.1186/1471-2164-11-218

**Published:** 2010-04-01

**Authors:** Alexander D Ball, Jessica Stapley, Deborah A Dawson, Tim R Birkhead, Terry Burke, Jon Slate

**Affiliations:** 1 Department of Animal and Plant Sciences, University of Sheffield, Sheffield, S10 2TN, UK

## Abstract

**Background:**

Genetic linkage maps are essential tools when searching for quantitative trait loci (QTL). To maximize genome coverage and provide an evenly spaced marker distribution a combination of different types of genetic marker are sometimes used. In this study we created linkage maps of four zebra finch (*Taeniopygia guttata*) chromosomes (1, 1A, 2 and 9) using two types of marker, Single Nucleotide Polymorphisms (SNPs) and microsatellites. To assess the effectiveness and accuracy of each kind of marker we compared maps built with each marker type separately and with both types of marker combined. Linkage map marker order was validated by making comparisons to the assembled zebra finch genome sequence.

**Results:**

We showed that marker order was less reliable and linkage map lengths were inflated for microsatellite maps relative to SNP maps, apparently due to differing error rates between the two types of marker. Guidelines on how to minimise the effects of error are provided. In particular, we show that when combining both types of marker the conventional process of building linkage maps, whereby the most informative markers are added to the map first, has to be modified in order to improve map accuracy.

**Conclusions:**

When using multiple types and large numbers of markers to create dense linkage maps, the least error prone loci (SNPs) rather than the most informative should be used to create framework maps before the addition of other potentially more error prone markers (microsatellites). This raises questions about the accuracy of marker order and predicted recombination rates in previous microsatellite linkage maps which were created using the conventional building process, however, provided suitable error detection strategies are followed microsatellite-based maps can continue to be regarded as reasonably reliable.

## Background

Linkage maps are fundamental tools in many genetic studies and have been created using various types of polymorphic markers since their conception by Sturtevant in 1913 [1]. Genetic linkage maps determine the linear position of genes or markers on a chromosome. They also provide information on genome wide recombination rates, as well as insight into intra and inter-species gene rearrangements within and between chromosomes; thus maps are useful in the study of evolutionary and comparative genomics [2-4]. However one of their most important applications is in the search for Mendelian and Quantitative Trait Loci (QTL) [5,6]. Over the last four decades advances in molecular technology have meant a wider range and greater number of genetic markers have become available, enabling linkage maps to be created for an increasing number of species, including many non-model organisms [7-9]. It is therefore timely to consider how different markers and different building approaches may influence the accuracy of genetic maps.

Two key components to consider when constructing a linkage map are the number and type of markers to use. Phenotypic (i.e. visible) markers were the first to be utilized, but now the choice has been extended to include a range of molecular markers including allozymes, Random Amplified Polymorphic DNA (RAPDs), Restriction Fragment Length Polymorphisms (RFLPs), Amplified Fragment Length Polymorphisms (AFLPs), Sequence-Tagged Sites (STSs), microsatellites (Simple Sequence Repeats) and SNPs. Each of these exhibit slightly different advantages and disadvantages but there are three main considerations when deciding which genetic markers to use in linkage map construction: (i) the markers need to be polymorphic, (ii) they need to be evenly spread across the genome or region of interest and provide dense marker coverage, and (iii) they must have a low genotyping error rate.

Microsatellites are obvious candidates for linkage mapping; they are highly polymorphic, relatively easy and cheap to score (once a library is established), and can exhibit cross-species utility in closely related species [10-13]. As a result they have been widely used in linkage mapping studies of humans, model organisms, agriculturally-important organisms and wild vertebrate populations [14-20]. Recently though, technological advances in locating and genotyping Single Nucleotide Polymorphisms (SNPs) has led to a decrease in both discovery time and genotyping cost [21,22]. This has caused an increase in their application in linkage mapping studies, exemplified in an updated high density chicken linkage map with 8599 SNPs [23] compared to the earlier map of 1889 molecular markers of which the majority were microsatellites [24]. This increased use of SNPs comes despite their (usually) biallelic nature, which means they provide relatively less information per locus. Lower variability means that they reveal fewer informative meioses, making linkage between markers harder to detect. To combat this, an increased number of markers, which are evenly spaced and cover a high proportion of the genome, can be used [25]. Additionally, it is possible to build maps with a combination of low-density, high-variability microsatellites and high-density, low-variability SNPs. The rationale of this approach is that the microsatellites might act as anchors and cause otherwise unassigned SNPs to be assimilated into linkage groups. This would produce linkage maps of greater accuracy or density, which will then be better suited to searching for QTL.

Microsatellites and SNPs also differ with respect to consideration (iii); the error rate. Markers with lower error rates obviously produce more accurate linkage data. A trade-off associated with the highly polymorphic nature of microsatellites is that they can have relatively high genotyping error rates [26,27]. Methods of genotyping and allele-calling microsatellites are only semi-automated, which can introduce human-based errors. Modern SNP genotyping platforms are almost fully automated and error rates tend to be much lower [28]. In previous studies much emphasis has been placed on identifying the consequences of genotyping error on the accuracy of population genetic analyses [29,30]. It has been acknowledged that these errors can impact on parentage inference or population structure analyses [26,27,31], but only recently has attention been focused on the extent to which genotyping errors can impact genetic linkage mapping [32,33]. In the human genetics literature, the relative merits of SNPs and microsatellite for genome-wide scans of complex diseases have been compared [34-38], but these studies have focused on the mapping of causative loci rather than the necessary initial step of map construction.

In a recent simulation study, where markers were compared for mapping accuracy, a difference in power between bi-allelic markers and polymorphic markers was reported, with a higher density of SNPs required to accurately produce similar results to the polymorphic microsatellites [32]. However, it was shown that when genotyping error rates are low, both SNP and microsatellite maps can be produced accurately. When a simulated 5% genotyping error rate was introduced to microsatellite genotyping, map inflation (sometimes by over 50%), incorrect marker order and even occasional allocation of markers to the incorrect linkage group occurred. This 5% level of error is not unprecedented in microsatellite genotyping, even when using invasive techniques of DNA acquisition [27,39]. Clearly there is a need to eliminate markers with genotyping errors ~5% wherever possible before proceeding with linkage analysis.

The aim of this study was to compare linkage maps built with microsatellites, SNPs and a combination of both markers to determine the ability of each method to produce accurate linkage maps. A linkage map of the zebra finch (*Taeniopygia guttata*) genome using SNP markers has recently been released [40], and a genome assembly is publicly available, which facilitates comparisons of marker order between linkage maps and physical assemblies. By independently constructing microsatellite linkage maps of the zebra finch macro-chromosomes 1, 1A, 2 and 9, using the same pedigree as was used in the SNP map, we were able to explore how marker type influenced map length and order. Guidelines for mapping studies that employ microsatellites and SNPs simultaneously are provided, as we recognise that sometimes it is practical to combine datasets containing both types of marker.

## Methods

The captive zebra finch population used in this study is one which has been maintained within the Department of Animal and Plant Sciences at the University of Sheffield since 1985 under the stewardship of TRB. The 3-generation mapping pedigree comprises 354 individuals and is the same pedigree as that used to build a SNP map of the whole zebra finch genome [40]. Genomic DNA was extracted from either blood or tissue samples using the ammonium acetate precipitation method [41]. The maintenance of the zebra finch pedigree along with the extraction of DNA was carried out in line with the UK Home Office guidelines under project license number 40/2788.

### SNP discovery and genotyping

The SNP genotyping data used in this study is the same as that previously published in [40]. This previous study was the first genome-wide zebra finch linkage map and incorporated 876 SNPs across 45 linkage groups. On the four chromosomes which are compared in our study there are 250 SNPs (73 on Tgu1, 71 on Tgu1A, 82 on Tgu2 and 24 on Tgu9). The SNPs were identified from cDNA sequences generated by Washington University Genome Researching Centre on a 454 Life Sciences ultra-high throughput pyrosequencing platform. The genotyping was undertaken by Illumina (San Diego) using the golden gate platform where 1356 potential SNPs were analysed. The genotyping data was estimated to be 100% reproducible, had a 95.5% call rate and a 0.17% parent-offspring error rate. After false and monomorphic SNPs and SNPs with a minor allele frequency <0.05 were discarded, 876 SNPs remained and were assembled into linkage groups. For a more detailed account of the SNP genotyping see [40].

### Microsatellite discovery

In order to discover polymorphic microsatellites, three methods were implemented. Initially, previously published microsatellites from the avian literature and a set of highly conserved microsatellite loci located in the zebra finch expressed sequence tag (EST) public databases were assessed [42,43]. By predicting chromosomal locations of the loci in the chicken genome sequence assembly [44], we discovered that the chromosomes 1, 2 and 9 had the highest number of already available microsatellites and so we subsequently focused on locating more microsatellites within these three chromosomes. However, in the zebra finch this equates to 4 chromosomes because Chicken chromosome 1 shares homology with two chromosomes in passerine birds [2,40,45,46]. Thus we were focusing on 4 chromosomes in the zebra finch (Tgu1, Tgu1A, Tgu2 and Tgu9).

We attempted to increase our marker density on these 4 chromosomes using the program SPUTNIK (Sputnik http://abajian.net/sputnik/) to search two zebra finch sequence databases for microsatellites. The first approach took advantage of the zebra finch EST sequences located on the National Centre for Biotechnology Information (NCBI) website (described in [47]), and the second searched the zebra finch Whole Genome Shotgun (WGS) GenBank tracefiles, as the assembled zebra finch genome was unavailable at that point. These searches were restricted to dinucleotide repeat motifs. In general, microsatellites with uninterrupted long repeats have been shown to be the most variable [48,49]. Therefore, only repeats of greater than 40 bp and with at least 90% purity were considered for further use. This ensured that microsatellites with the greatest potential for high variability were selected. As there can be more than one trace file for any region of the genome, files containing overlapping sequence were combined into contigs using the CAP3 contig assembly program [50].

A zebra finch homologous location for each microsatellite marker was found by directly comparing its sequence against the zebra finch genome assembly. Chromosomal locations were identified by Basic Local Alignment Search Tool (BLAST) analysis to compare the avian microsatellite or the zebra finch sequence (Whole Genome Shotgun (WGS) tracefiles or ESTs) against the zebra finch genome sequence (version 1, Zebra Finch Genome Consortium). BLAST analyses were carried out using Standalone BLAST version 2.2.17 [51] using the default parameters with minor modifications (W = 10, m = 8, e = 1e^-5^). The chromosome position with the lowest e-value was considered a good hit if the e-value of the hit was less than 1e^-10 ^and the difference between it and the next best hit was 10 decimal places. In general, the length of the query sequence significantly affects whether a reliable BLAST hit can be found [3]. However, the zebra finch WGS sequence trace files and ESTs were usually about 600 bp long and thus produced reliable hits on the genome with e-values of much less than 1e-5 (See additional file 1: Table S1, for characterization of the microsatellites). These positions allowed us to focus our attention on the loci located only on the chromosomes of interest (Tgu1, Tgu1A, Tgu2 and Tgu9).

### Microsatellite primer design and testing

There are three minor distinctions between the way loci were identified and primers designed in this study. These can be discerned by the names used for each primer pair, with the prefixes ZF, TG and ZEST used to discriminate between the different design methods (Table 1).

**Table 1:** Summary of genotyping results.

Locus	Locus reference	Number of individuals genotyped in study population	Predicted allele size	Observed allele size range	Number of alleles obs.	H_e_	H_o_	Est. null allele freq. (CERVUS v3.0)	Mean estimated error rate (CERVUS v3.0)	Multiplex set
ZF01-020	This study	320	178	160-261	15	0.69	0.59	0.075	0.007	3
ZF01-025	This study	334	211	188-217	11	0.78	0.77	0.005	0	2, 3
TG01-040	This study	307	286	286-294	7	0.69	0.70	-0.011	0	2
ZF01-054^a^	This study	308	185	157-188	13	0.89	0.75	0.083	0.002	2
ZF01-139^b^	This study	330	188	156-209	4	0.53	0.55	-0.007	0.040	4
ZF01-136	This study	299	367	333-373	10	0.73	0.55	0.138^$^	0	2
ZF01-170^c^	[47]	21	316	257-305	9	0.83	0.86	-0.032	-	4
ZF01-161	This study	333	159	125-168	8	0.70	0.74	-0.036	0	4
ZF01-190	This study	329	342	308-366	12	0.83	0.87	-0.026	0	3
ZF01-196	This study	334	292	270-304	7	0.74	0.77	-0.014	0	2, 3, 4
Tgu12	This study	331	282	248-273	10	0.79	0.75	0.026	0.016	3
ZF01-180^b^	[43]	331	213	166-218	8	0.76	0.66	0.071	0.060	4
ZF01-081^b^	This study	337	233	130-154	5	0.55	0.51	0.031	0.077	4
ZF02-068	This study	292	185	121-152	7	0.70	0.50	0.157^$^	0.010	2
ZF02-038^b^	This study	301	233	211-245	11	0.77	0.66	0.074	0.085	2
TG02-078	[43]	314	309	308-321	6	0.73	0.76	-0.024	0	1
TG02-088	[43]	310	268	263-269	5	0.73	0.77	-0.034	0	1
Ase44	[64]	303	268	308-329	8	0.81	0.79	0.010	0.006	2
ZF02-128	This study	301	374	365-435	9	0.83	0.79	0.022	0.005	2
ZF02-129	This study	329	169	124-172	11	0.83	0.82	0.004	0.005	3
ZEST09-005	Dawson et al. unpublished	316	168	155-165	5	0.74	0.67	0.047	0	1
Smm4	[65]	313	332	332-341	6	0.57	0.50	0.049^$^	0.018	1
Cpi7	[66]	308	128	119-131	5	0.54	0.55	-0.016	0	1
ZEST09-018	Dawson et al. unpublished	289	285	282-293	8	0.70	0.75	-0.039	0	1
ZEST09-021	Dawson et al. unpublished	311	119	114-121	5	0.47	0.41	0.096	0	1
ZEST09-025	Dawson et al. unpublished	281	167	164-170	6	0.71	0.69	0.014	0	1

Information is for the 26 zebra finch (*Taeniopygia guttata*) microsatellite markers used to create the linkage maps for the zebra finch chromosomes 1, 1A, 2 and 9. See additional file 1: Table S1 for more information.

H_e_, H_o _expected and observed heterozygosity (calculated using CERVUS v3.0)

$, Markers with null alleles; null alleles were detectable by following their segregation through the pedigree. Null alleles were rescored as allele 99 before running through CriMap. Null allele frequencies are calculated using the original genotypes and are based on the excess of homozygous individuals.

a, Excluded after CHROMPIC revealed an excess of double recombination events with adjacent markers, indicative of high error rate.

b, Excluded from linkage maps as parent-offspring mismatches estimated the error rate > 0.02

c, Could not be accurately scored after multiplexing.

First, to increase the probability of microsatellite co-amplification in other species, consensus sequences were used to identify the most conserved regions for primer design. For these markers, prefixed "TG", the primer sites are in regions of 100% identity between the zebra finch and chicken (their design is described elsewhere [43]). Second, primers for markers with prefix "ZF" were created using only the zebra finch sequence from the WGS sequence trace files. Finally, markers labeled ZEST were designed from sequences first reported in [47] and are microsatellites isolated from the zebra finch EST Genbank entries. Primers were designed using these zebra finch EST sequences rather than genomic sequence.

Primers were designed using the web interface of the program PRIMER3 ([52], http://frodo.wi.mit.edu/primer3/). The PRIMER3 default parameters were used, except that a CG clamp was required at the 3' end of each primer and the maximum consecutive number of the same bases (Max-polyX) was lowered to 3. The maximum primer melting temperature (Tm) was set at 60°C, the minimum was set at 54°C and the maximum Tm difference between the forward and reverse primer was lowered to 0.5°C. By using the settings stated above we attempted to ensure all primers would amplify with a Polymerase Chain Reaction (PCR) annealing temperature of 56°C.

The forward primer was labeled with a fluoro-dye (6-FAM, 5HEX or NED) to enable allele size assignment on an ABI 3730 DNA Analyzer. The initial tests for amplification were each carried out in 10 μl PCR reactions. These contained about 50 ng of genomic DNA, 1.0 μM of each primer and 0.25 units Taq DNA polymerase (ThermoprimePlus, Advanced Biotechnologies) in the manufacturer's buffer (final concentrations 20 mM (NH_4_)_2_SO_4_, 75 mM Tris-HCl pH 9.0, 0.01% (w/v) Tween), including 2.0 mM MgCl_2 _and 0.2 mM of each dNTP. PCR amplification was performed in a TETRAD DNA Engine (MJ Research, Biorad) or a Touchdown thermal cycler (Hybaid). The following PCR program was used for these singleplex test reactions, 3 minutes at 94°C, followed by 35 cycles of 30 seconds at 94°C, 30 seconds at 56°C and 30 seconds at 72°C, then 10 minutes at 72°C. PCR products were then separated and visualized using an ABI 3730 DNA Analyzer (Applied Biosystems) using Prism set DS-30 and the ROX size standard (ABI, Foster City, USA) and allele sizes assigned using GENEMAPPER 3.7 (Applied Biosystems).

Sixty-four microsatellite primers were initially tested for PCR amplification success and polymorphism. Of these, 26 were first tested on 12 zebra finch individuals (4 wild zebra finch and 8 mapping pedigree individuals). Any markers which had more than 2 alleles were then tested on 24 birds in the mapping population. However, the 38 primers with prefix "ZF" were initially tested in all 24 individuals. After this, observed and expected heterozygosity and predicted null allele frequencies were estimated using CERVUS v3.0 [53]. A total of 26 markers displayed more than 3 alleles and had an observed heterozygosity greater than 0.3, and were then used for further analysis. The zebra finch mapping pedigree containing 354 individuals was genotyped at these 26 loci (Table 1). Unfortunately, one of these markers (ZF01-170) failed to amplify correctly within its multiplex set.

### Microsatellite genotyping and error rate checking

The majority of the pedigree individuals were of known phenotypic sex (171 females, 175 males and 8 individuals of unknown sex). All individuals were PCR sex-typed using the Z-002 sex-typing primers [54] during the genotyping to check for any identity errors. There were two cases of mismatches between genotypic and phenotypic sex; these samples, along with eleven others, contained inheritance errors, exhibiting incompatible genotypes with known relatives. They were excluded from further analysis.

When genotyping the whole pedigree, a Qiagen multiplex mix was used during PCR amplification. Four multiplex sets were designed, each containing between 6-9 markers (Table 1). All markers in each set were co-amplified and loaded onto an ABI 3730 DNA Analyzer. The PCR program was the same as used in the singleplex reactions except the annealing temperature (Ta) was increased to 57°C; see Qiagen multiplex guidelines for the rest of the protocol.

Two markers (ZF01-196 and ZF01-025) were included in multiple multiplex sets to verify repeatability between the different PCR sets. Marker ZF01-196 was multiplexed in sets 2, 3 and 4 and ZF01-025 in sets 2 and 3. For ZF01-196 the number of samples that did not amplify was significantly greater in set 2 (42) than in sets 3 (10) and 4 (18) (χ^2 ^= 23.8, p < 0.005, d.f. = 2). This was also the case for ZF01-025 with set 2 containing 61 ungenotyped samples compared to 24 in set 3 (χ^2 ^= 16.1, p < 0.001, d.f. = 1). The lower call rate in Set 2 is probably due to the increased number of markers incorporated into this set, as increased competition between primers could lead to reduced amplification of some loci. In total, 12 of the 273 individuals, which could be genotyped at locus ZF01-196 in all 3 sets, exhibited 2 different genotypes. The genotype that was exhibited in 2 of the sets was assumed to be the correct one, which gave a 1.46% error rate at this locus. The repeatability of locus ZF01-025 was better with only one genotyping inconsistency within the 289 samples that were genotyped in both sets giving an error rate of 0.17%. The errors were distributed across all multiplex sets, making it difficult to identify the reasons for these anomalies, but suggesting that they were not due to consistent differences between the quality or reliability of the multiplexes.

An alternative method of checking the error rate is to measure the frequency of deviations from Mendelian inheritance. After genotyping the full pedigree we used the programs PedCheck and CERVUS v3.0 to assess the quality of the data [55,56]. Where parent-offspring genotype mismatches were identified we reanalysed the 3730 output in GENEMAPPER. In most cases an allele had been missed or incorrectly genotyped and was therefore corrected. After this, CERVUS was used to estimate null allele frequencies, and the remaining parent offspring mismatches were used to estimate the error rate. For some markers with high estimated null allele frequencies it was possible to identify where null alleles were segregating in the pedigree. In order to keep as many informative recombination events as possible in the analyses, these null alleles were dummy-coded as allele '99' (for markers ZF01-136, ZF02-068 & Smm4(ZF09-004)). These data could then still be used to create the linkage maps. Any markers which had a parent-offspring error rate of >2% were excluded from further analysis (4 markers) as it has been shown by simulation that typing error can cause map inflation and affect marker allocation to linkage maps [32]. In total the number of microsatellite markers initially used to create linkage maps of chromosomes Tgu1, Tgu1A, Tgu2 and Tgu9 were 5, 4, 6 and 6 respectively (Table 1).

### Map construction

Linkage maps were constructed using a version of CriMap 2.4 [57] modified by Xuelu Liu (Animal Genomics and Breeding group, Monsanto Company) to accommodate large numbers of markers segregating in complicated pedigree structures. Initially, the pedigree was split into 14 sub-families using the CRIGEN function. Then the TWOPOINT command was used to calculate the two-point LOD scores between all possible marker pairs. AUTOGROUP was implemented to assign the markers to linkage groups. AUTOGROUP groups markers via an iterative process based on marker variability and linkage quality, starting at an upper, stringent layer and proceeding through lower, less stringent layers. The parameter layers were as follows: Layer 1 (40, 2.0, 2, 0.9); Layer 2 (20, 1.5, 3, 0.7); Layer 3 (10, 1.0, 5, 0.6); Layer 4 (5, 0.4, 6, 0.5). The lower layer defines the minimum requirements for inclusion in a linkage group. In this way, linkage groups were created between markers that were linked with a two-point LOD score > 5, had a minimum of 0.4 times the average number of meioses, shared linkages with no more than six other groups and had a minimum linkage ratio (i.e. the proportion of two point linkages for a given marker to other markers in the same linkage group) of 0.5. Once the markers were assigned to linkage groups relative marker order was determined as described below.

During the initial stages of map building redundant markers were removed using the HAPLOGROUP command. This identified tightly linked markers (recombination fraction = 0, LOD >10) and used only the most informative marker in each haplogroup for map assembly. Using the remaining informative markers, framework maps were created using the BUILD command. The BUILD command was implemented by entering two linked, informative markers with recombination fractions of between 0.1-0.2 as ordered loci, and inserting the remaining loci. The likelihood threshold was set at 5, so the marker order with a likelihood of LOD ≥ 5 better than the next most likely order was chosen. The process was repeated twice, each time starting with a different pair of markers. Of the three maps, the one with the most markers was chosen as the first framework map. The FLIPS5 function was used to shuffle marker orders and identify the most likely marker order. Using this framework map, we added the remaining markers by progressively dropping the likelihood threshold to LOD = 3, 1, 0.1, 0.01 and then 0.001. This results in a comprehensive map of all markers, with the final order determined by the best likelihood given the data available. The positions of markers mapped at the lower LOD thresholds are less well supported. In most studies a LOD of ≥3, i.e. marker order 1000 times more likely than any other marker order is considered a conservative (or framework) map [58]. The FLIPS5 command was again used to check that no alternative order had a higher likelihood. Next, the markers previously excluded (via the HAPLOGROUP command) were added to the assembled map using BUILD at a likelihood threshold of LOD > 0.001 and marker order was again rechecked using FLIPS5. Finally the command CHROMPIC was used to identify any putative double recombination events between closely linked markers that may be indicative of incorrect marker order or genotype errors. CHROMPIC provides a schematic of the paternally and maternally inherited chromosomes of each individual, with the inferred grandparental origin of each marker allele indicated. This schematic shows each recombination event and flags any alleles which do not share the same grandparental origin with alleles at adjacent markers on the same haplotype. If one marker is flagged in a large number of individuals this suggests that it is either placed in the wrong location on the linkage map or has a high error rate.

Using CHROMPIC, one microsatellite marker (ZF01-054) was excluded from Tgu1A as even when positioned in its location with highest likelihood it was consistently flagged, suggesting it had a high error rate. This was also supported by the fact that its presence increased map length by a substantial margin (20 cM) [32]. A subsequent BLAST of the zebra finch trace file used for the primer design against the released zebra finch genome sequence, revealed that the first two bases at the 5' end of the forward primer were not consistent with the genome assembly sequence. This could potentially cause allelic drop-out leading to the high degree of error that is likely present in the genotypes at this locus. This exclusion reduced the number of microsatellites on this linkage group to three. All linkage map distances are expressed in Kosambi centiMorgans (cM) and the map figures were constructed in MapChart [59].

### Examining map accuracy

Linkage maps were constructed for the microsatellites and the SNPs separately using the approach outlined above. We also built combined linkage maps including both the microsatellites and SNPs. We constructed these combined maps via two different methods. The first approach followed the conventional method as described above and elsewhere [40]. Typically, this would mean microsatellites would be among the first markers added to the map, as they are highly informative. Hereafter the map is termed the 'SNPs & microsatellites conventional' map. The second approach involved adding the relevant microsatellites directly to the comprehensive SNP linkage maps (presented in [40]). This was carried out using BUILD, and as with previous approaches the likelihood threshold was progressively reduced (LOD ≥ 5, 3, 1, 0, 0.05, 0.001) until all the microsatellites were added to the linkage map. Here the microsatellites were added *after *the SNPs (hereafter this is termed the 'SNPs preceding microsatellites' map).

To validate the accuracy of the maps we compared marker order on each linkage map with the order on the zebra finch genome assembly http://genome.wustl.edu/genomes/view/taeniopygia_guttata/. We also compared SNP-only maps with maps that contained SNPs and microsatellites, in order to test whether the inclusion of microsatellites caused map length inflation.

## Results & Discussion

### Error rate

Microsatellite genotyping error has been highlighted as a major cause for concern in population genetic analyses, including parentage assignment (reviewed in [27]). Particular attention has been given to studies using non-invasive techniques for DNA acquisition, but errors are present in all studies and this is increasingly being recognised in the literature [60]. Even in studies dealing with high quality DNA samples, the potential for error remains, particularly those caused by human error. When one investigation critically assessed the degree of genotyping error in an Antarctic fur seal paternity exclusion study, up to 93% of the errors were attributable to human mistakes [26,27].

The greater number of alleles of microsatellite markers, along with a less automated method of genotyping leads to a higher error rate relative to SNPs [25]. Within the SNP mapping project [40] one individual was repeatedly genotyped 7 times at all 1048 SNPs to measure repeatability, and no errors were detected (100% accuracy). Also, across all SNPs the average parent-offspring mismatch rate was 0.17%. In contrast, for the 25 microsatellites initially genotyped in the zebra finch mapping pedigree the mean error rate per locus was 1.32%, based on the frequency of departures from Mendelian inheritance implemented in CERVUS (Table 1). This value is ~7 times more than the SNP error rate. For construction of the maps, only microsatellite markers with an error rate <2% based on parent-offspring mismatches were used. As a result, four markers (ZF01-139, ZF01-180, ZF01-081 & ZF02-038) were excluded from further analyses. For the microsatellites used in the final linkage maps the average error rate was 0.34% based on Mendelian inheritance inconsistencies. This is fairly low, although it is still almost double that found in the SNPs, even though the most error prone microsatellites have been removed from the dataset while all SNPs were retained.

Although genotyping errors are not uncommon, most can be easily identified. In this study the pedigree was known, and due to the number of markers typed in the entire pedigree (25), it was possible to detect any allele calls that caused departures from Mendelian inheritance. Any individuals involved in a parent-offspring mismatch were re-examined on the program GENEMAPPER. Eleven individuals were inconsistent with Mendelian inheritance at greater than 5 microsatellite loci and therefore were assumed to have been contaminated or incorrectly labelled. The results from these samples were subsequently excluded from all analysis. The high variability of microsatellites aids error detection as most errors will result in Mendelian incompatibilities. However, with SNPs this error-checking procedure will be less successful as they are only biallelic. Some studies have predicted that up to 30% of errors in SNP genotyping will remain undetected because they will not cause Mendelian inheritance incompatibilities [61,62].

After re-examining the allele calling any remaining mismatches could be due to allelic dropout, scoring error, more cryptic biochemical artefacts or *de novo *mutations in the germline. Given that we have excluded loci with high error rates, identified mis-labelled or mis-pedigreed individuals and re-scored individuals that cause mismatches or unlikely double recombination events, it seems reasonable to assume that the subsequent impact of typing error on map construction that we report is conservative. Of course, it should be regarded good practice to remove as much of the detectable error as possible. It is important to note, and perhaps underappreciated, that some genotype errors will not cause Mendelian inconsistencies, but can still lead to the inference of spurious recombination events. For example, suppose an offspring with a true genotype of A/A was miss-scored as A/B. If it has parents with genotype A/A (mother) and A/B (father) both the true and incorrect genotype are compatible with Mendelian inheritance. However it would be wrongly assumed that allele B was inherited from the father, which would lead to the inference of at least one, but more likely two, additional recombination events. Therefore, even with careful error checking, loci with high error rates are likely to cause map inflation.

### Robustness and reliability of maps

#### (i) Assignment of markers to the correct chromosome

The linkage maps generated by the 4 different methods are presented in additional file 2: Table S2, along with the physical positions of each marker on the zebra finch genome assembly. As anticipated, both SNP and microsatellite markers formed linkage groups corresponding to their predicted chromosome based on the zebra finch assembly. We have only presented the sex-averaged linkage maps as there is little evidence of heterochiasmy in the SNP linkage maps [40].

**Figure 1:**
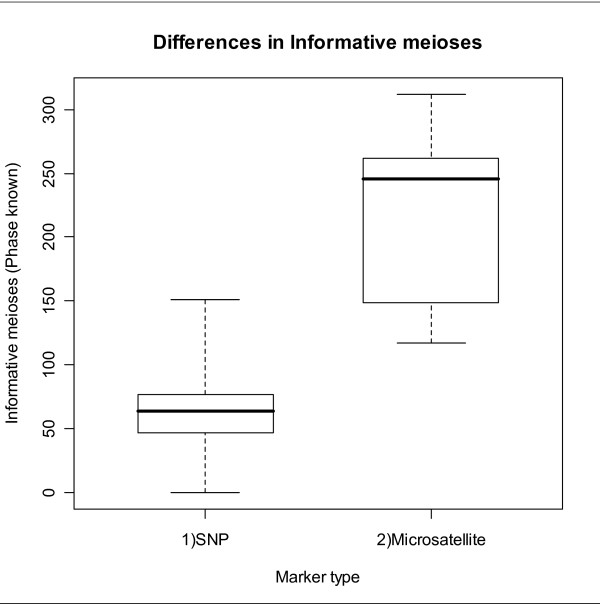
**Number of informative meioses**. Comparison of the number of informative meioses of microsatellites and SNPs. Informative meioses are highly dependent on the variability of the marker. The microsatellite markers exhibit significantly more informative meioses than the SNPs (t-test, t = 10.71, p < 0.0001(two-tailed), d.f. = 19.44).

The greatest difference between the SNP and microsatellite maps is the number of markers on each, with all chromosomes containing substantially more SNPs. The linkage groups Tgu1, Tgu1A, Tgu2 and Tgu9 contain 73, 71, 82 and 24 SNPs respectively compared to only 3, 5, 6 and 6 microsatellites. However, every microsatellite was assigned to the correct linkage group, even on the low-density microsatellite only maps. The ability to detect linkage between the microsatellites when so few are used is due to the relatively high number of informative meioses of these markers (Figure 1). Across all 265 markers, the number of phase known informative meioses was significantly greater for the microsatellites (mean = 216.3) than for the SNPs (mean = 62.3; t = -10.71, p < 0.0001 (two-tailed), d.f. = 19.44).

The number of markers used in linkage maps obviously impacts on the coverage that the markers provide over the chromosomes. In all cases the SNPs provide a higher coverage of the 4 focal chromosomes. For linkage groups Tgu1, Tgu1A, Tgu2 and Tgu9, about 76%, 96%, 73% and 90% respectively of the chromosomes are estimated to be within 2.5 Mbp of a SNP marker. However, the coverage for the same chromosomes is only 24%, 20%, 16% and 76% for the microsatellite markers.

#### (ii) Estimation of recombination rates and distribution of recombination events

**Figure 2:**
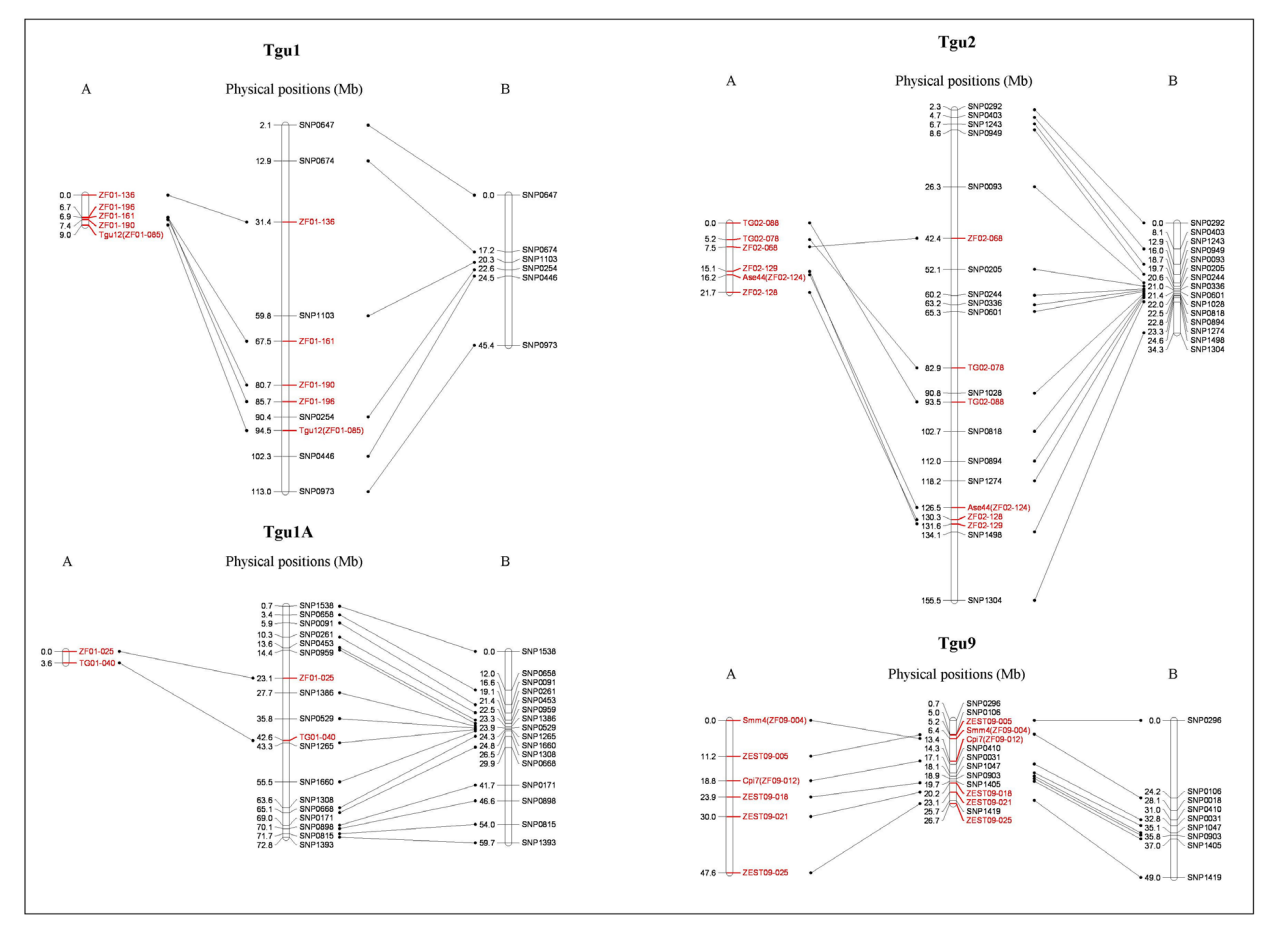
**Separate SNP and microsatellite framework maps**. Linkage maps built via the conventional method show that (A) marker order of microsatellites maps does not always correspond to the physical order on the zebra finch genome assembly (centre). However, for all four chromosomes the order of maps built with only the SNP markers (B) does agree with the zebra finch assembly order. Framework maps with marker order supported by LOD ≥ 3 are presented. Microsatellites are highlighted in red and linkage map positions are given in cM (A and B). The predicted genome assembly positions for the markers (centre) are given in Mb.

As previously highlighted [40,63] and observed to a degree on the framework SNP maps in Figure 2, recombination rates in the zebra finch appear greatest near the telomeres of the chromosomes, especially on the macro-chromosomes. Most of the microsatellite markers were not positioned near to the end of the chromosomes and so are predicted to be in regions with lower recombination rates. Therefore, to compare recombination rates between the SNP and microsatellite maps, it was necessary to restrict comparisons to regions on the SNP map that overlap the chromosomal regions covered by the microsatellites.

The microsatellite maps consistently estimated higher recombination rates than did the SNP maps. For example, on Tgu1A the microsatellites span a predicted 24.4 Mbp and the linkage distance is 15.8 cM, whereas a similar region covering 21.7 Mbp is only 0.6 cM on the SNP map. On linkage group Tgu1 (region spanning ~31.4 Mbp to 94.5 Mbp) the microsatellite map exhibits a recombination rate (0.143 cM Mb^-1^) more than 7 times greater than the SNP map (0.02 cM Mb^-1^). The Tgu2 maps are more difficult to compare as both comprehensive maps exhibit rearrangements compared to the genome sequence (Additional file 2: Table S2). For these we used the distal microsatellites according to the physical chromosome positions and used the whole of the genetic map length to estimate the recombination rate. As the marker order with the highest likelihood is typically shorter than any alternative order this should give a conservative estimate. The microsatellite recombination rate (0.24 cM Mb^-1^) was ten times greater than that predicted by the SNP map (0.02 cM Mb^-1^). This degree of inflation is worrying, especially considering the attempts to control for the influence of microsatellite typing error. For Tgu9 we also estimated the recombination rates using the distal microsatellites according to the genome assembly. The maps lengths were more similar but the recombination rate of the microsatellite maps was still inflated (microsatellite map = 2.22 cM Mb^-1^, SNP map = 1.75 cM Mb^-1^). In general, the difference between SNP and microsatellite recombination rates seems to be most pronounced in the central parts of macro-chromosomes. This is logical, as recombination in these regions is relatively rare, and therefore the relative impact of spurious recombination events caused by typing error will be greater than in regions where true recombination events are more frequent.

**Figure 3:**
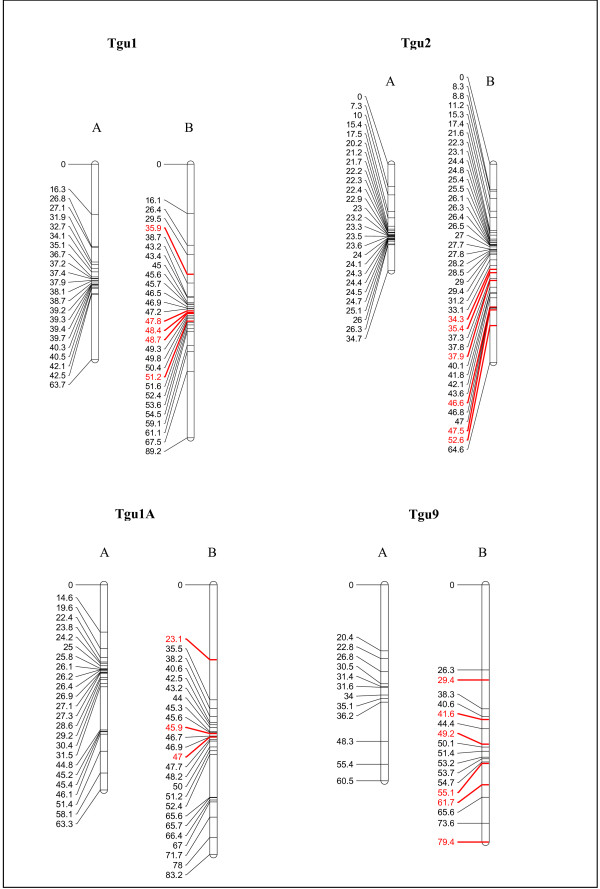
**Inflation caused by adding microsatellites**. Linkage maps created with (A) only the SNP markers are substantially shorter in length than (B) linkage maps created using both SNP and microsatellite markers when the conventional build method is used. This inflation is exhibited in all four chromosomes. Generally, the microsatellites (highlighted in red) are not distal to the SNP markers so the cause of the inflation is likely to be genotyping error at the microsatellite markers.

The degree of map inflation caused by microsatellites was reduced, but still noticeable, when microsatellites and SNPs were combined via the conventional build method. In general, maps built from microsatellites and SNPs were longer than SNP-only maps, even though the microsatellites were only distal to the SNPs in the Tgu9 map (Figure 3). Tgu1: SNP map = 63.7 cM; SNP and microsatellite map = 89.2 cM; Tgu1A: SNP map = 63.3 cM, SNP and microsatellite map = 83.2 cM; Tgu 2: SNP map = 34.7 cM, SNP and microsatellite map = 64.6 cM; Tgu 9: SNP map = 60.5 cM, SNP and microsatellite map = 79.4 cM.

One conceivable explanation for the differences between the microsatellite and SNP maps is that the proportion of missing data was three times greater for microsatellties (15%) than SNPs (5%). This is unlikely to lead to map inflation of the microsatellite maps as there is no reason why missing genotypes should be biased with respect to whether they affect recombinant or non-recombinant meiotic events. To test this further we simulated 10 replicates of the LG1 SNP map (63.7 cM) with 15% missing data and then rebuilt the maps. The simulated datasets had a mean map length almost identical to the original (mean length = 64.1 cM; SE 0.9 cM). In other words there was no evidence that map inflation of the microsatellite maps was caused by a higher genotyping failure rate. It is also worth noting that the greater variability of microsatellites means that they resulted in a much higher number of informative meioses than the SNPs (see Figure 1), which more than offsets the higher failure rate.

The most likely explanation for the discrepancies in the map lengths is the higher typing error rate of the microsatellites. It seems even after carefully checking for errors and excluding error prone markers, recombination rate appears inflated. This could be a phenomenon in many microsatellite based linkage maps and needs to be considered when comparing linkage maps constructed from different types of marker. Recently, a similar conclusion was reached in a paper that combined SNP, microsatellite and AFLP data to build a linkage map for the chicken genome [23]. During construction of this high density linkage map, 70% of the previously mapped markers (the majority of which were microsatellites) had to be excluded from the updated map (which mostly contained SNPs), as they caused map inflation, despite rigorous error checking when the microsatellite map was first constructed [24]. This phenomenon could be prevalent for many previously created microsatellite linkage maps and it seems likely that subsequent high density SNP maps will reveal many medium density microsatellite maps to be inflated.

#### (iii) Accuracy of marker order

To validate the marker order of SNP and microsatellite maps, the zebra finch genome sequence assembly was used as a template against which all linkage maps were evaluated. It is important to note that the SNP map has been used as a reference during the latter part of the zebra finch sequence assembly process (for a more detailed account of the zebra finch assembly process see http://genome.wustl.edu/pub/organism/Other_Vertebrates/Taeniopygia_guttata/assembly/Taeniopygia_guttata-3.2.4/). However, the degree to which this has influenced the order of markers on the physical map is thought to be negligible. As a precaution we have also used assembled supercontigs as a reference against which to compare orders of the different linkage maps (see below). These supercontigs were created independently of any linkage map and so provide a robust test of the accuracy of map order, without any potential influence of the linkage map on the assembled zebra finch sequence.

Very few of the comprehensive maps are in complete agreement with the assembled genome sequence. It is only the microsatellite map for Tgu1A and the SNP map for Tgu9 that adhere completely to the sequence order. This is not surprising, as by definition, the comprehensive map may have a marker order with only marginally stronger statistical support than the next most likely order. Marker orders supported by a LOD <3.0 are not generally regarded as definitive. Therefore, it is more revealing to examine framework maps where the best order is supported by a LOD ≥ 3 and is assumed to be reliable. Here all the SNP maps are in complete agreement with the genome assembly, but the order of three of the four microsatellite maps differs from the genome assembly (see Figure 2); the exception is Tgu1A, which only includes 2 markers. This would indicate that even the most conservative microsatellite maps are unreliable. For example, ZF09-004, (estimated null frequency = 0.049, estimated error rate = 0.018) causes an apparent inversion (relative to the physical assembly) on linkage group Tgu9 which is not supported by the SNPs. Even after exclusion of errors and selection of markers with less than 2% error rates, the microsatellite linkage maps are not only inflated, but also exhibit different marker orders to the known sequence.

In general, the SNPs-only and 'SNPs preceding microsatellites' maps provide a closer correspondence to the genome assembly than the microsatellites-only and the 'SNPs & microsatellites conventional' maps (Additional file 2: Table S2). Examination of the framework maps (marker order supported by a LOD ≥ 3) reveals that the 'SNPs preceding microsatellite' (Additional file 2: Table S2) framework maps correspond to the genome assembly for every chromosome, whereas the microsatellite-only and the 'SNPs and microsatellites conventional' framework maps generally do not (Additional file 2: Table S2). The reason for these discrepancies is likely to be typing error or null alleles at the microsatellites, which have previously been found to inflate linkage maps and support incorrect marker orders [32].

It could be argued that because the SNP map was used to help assemble the zebra finch genome its errors could have been incorporated into the physical assembly, thereby making the assembly an inappropriate reference against which to compare the microsatellite and SNP maps. To address this issue we compared the order of markers in the "SNP only" and "Microsatellite only" maps against the supercontig sequences which were created independently of the linkage maps. These are assembled genome sequences (n = 37,698) with an average length of ~10 Mb. For each framework marker (Figure 2) the supercontig location (both the supercontig name and position within that supercontig) was obtained via a BLAST search of the zebra finch genome. Any supercontigs that contained either ≥3 framework SNPs (4 contigs) or microsatellites (1 contig) were used to investigate map accuracy by comparing the order of markers on the linkage maps and supercontigs. For all supercontigs containing ≥3 framework SNPs (Contig22 = 5 SNPs, Contig 2 = 4 SNPs, Contig43 = 3 SNPs and Contig41 = 3 SNPs) the marker order was identical to the "SNP only" linkage map. However, the 3 framework microsatellites with BLAST positions on Contig 2, were ordered Ase44 - ZF02-128 - ZF02-129 on the supercontig and ZF02-129 - Ase44 - ZF02-128 on the "Microsatellite only" linkage map. This further demonstrates the inability of the microsatellite maps to infer the correct order of markers.

It should be acknowledged that some discrepancies between the linkage maps and the physical genome sequence could be indicative of mistakes in the assembly. Genome assemblies are an ongoing process, and it is likely that the zebra finch assembly will be improved as more information becomes available, and possibly as the chicken genome assembly improves. Limitations of the zebra finch sequence assembly were apparent when performing BLAST searches; four SNP markers (SNP0444, SNP0154, SNP0853 & SNP0018) had hits on the chicken genome assembly to the chromosomes predicted by the linkage maps, yet they fail to produce a hit on the zebra finch genome sequence. As these SNPs have been isolated and obtained from zebra finch DNA, it suggests that the zebra finch genome assembly remains relatively incomplete. Putative assembly errors however, cannot explain the much greater recombination rates of the microsatellite maps, and there is no a priori reason to expect inaccuracies in the assembly to affect marker order of microsatellites but not SNPs.

Another key point to consider when assessing these maps is that the greater marker density of the SNP maps, make the detection of 'unusual' double recombinants indicative of typing error more obvious. The problems associated with microsatellites highlighted in this paper may be less pronounced if more markers were typed, although they are unlikely to disappear completely as evidenced by the map inflation of the high density combined SNP and microsatellite maps, and by the previously undetected map inflation observed on the high density microsatellite-based chicken linkage maps [23].

#### (iv) Comparisons of the 'SNPs and microsatellites conventional' and 'SNPs preceding microsatellites' maps

**Figure 4:**
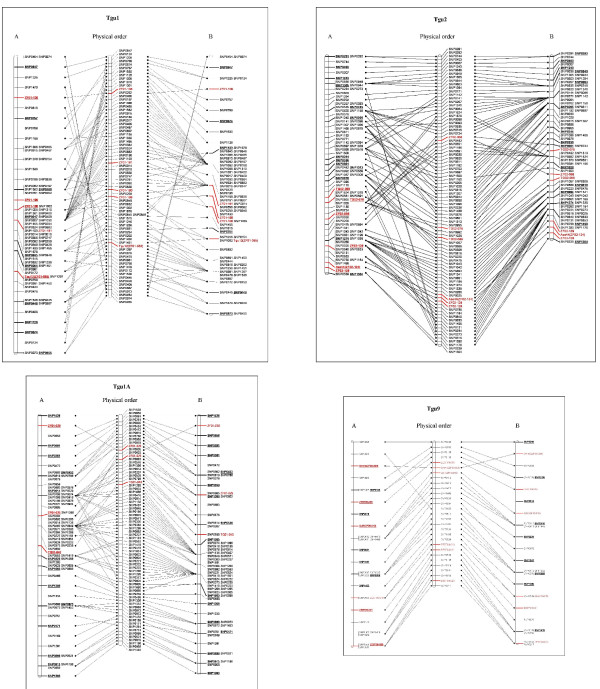
**Comparison of two methods of building combined microsatellite and SNP maps**. (A) The maps created by the conventional method. (B) Maps created by the method where the SNP markers are mapped before the microsatellites. The maps produced by the 'SNPs preceding microsatellites' are more similar to the physical order of the markers in the genome assembly, which are shown in the centre. The microsatellite markers are highlighted in red and the markers positioned at a LOD ≥ 3 are in bold, underlined font. To aid clarity, the markers on the linkage maps are shown at equidistant positions along the chromosomes, although the total map lengths are shown on the same scale, illustrating the inflated lengths of the conventional build process.

When combined, the microsatellites and SNPs were assigned to the predicted linkage groups based on their known chicken and zebra finch assembly positions and their earlier linkage map positions [40]. Interestingly, the two different methods used for building maps gave quite disparate map lengths (Figure 4, Additional file 2: Table S2). The linkage maps built via the conventional method, which typically means microsatellites are among the earliest to be added to the map, were longer than the equivalent maps that were constructed by adding microsatellites to the SNP maps (Tgu1 11.8 cM longer, Tgu1A 0.9 cM longer, Tgu2 10.2 cM longer, Tgu9 1.0 cM longer). Furthermore, maps built by the conventional method had marker orders supported by a lower likelihood (Tgu1 35.5 lower, Tgu1A 7.5 lower, Tgu2 87.0 lower, Tgu9 9.6 lower) and showed more inconsistencies with the zebra finch assembly order, suggesting that the conventional method was more error prone than when mapping the SNPs first (Figure 4, Additional file 2: Table S2).

It is likely that the combination of greater informativeness and higher errors of the microsatellites cause them to be added first but in the wrong order. Subsequent addition of the SNPs fails to rectify the errors as the relatively large number of SNPs (compared to microsatellites) renders the FLIPS5 command unable to resolve errors in marker order between microsatellites separated by large numbers of SNPs. FLIPS takes consecutive markers, reorders them and produces likelihood scores for these different orders. Due to computational constraints it is impractical to use FLIPS for greater than five markers. What can happen in this situation is that a local likelihood maxima is reached between a group of closely linked markers such that no changes in the order of these will give a better likelihood. However, if it was possible to flip the order of more than 5 markers then a more accurate order may be revealed.

One of the outcomes of this investigation is that the conventional method of building linkage maps has to be modified when using markers that differ significantly in their number of informative meioses and error rates. Markers with the lowest error rates should be included first to provide an accurate foundation, to which other markers can then be added. This may be especially important for larger chromosomes as Tgu1, Tgu1A and Tgu2 exhibited the largest differences between the two construction methods, whereas the smaller chromosome Tgu9 did not differ as substantially. It is possible that those regions of macro-chromosomes with very low recombination rates are most sensitive to typing error. However this could also be due to the markers on Tgu9 exhibiting lower error rates, and investigation of additional smaller chromosomes would be needed to confirm this observation.

## Conclusions

This study highlights potential problems, as well as some solutions, of linkage mapping construction with moderately error prone markers. Although microsatellites are informative, they can provide misleading results because they have greater error rates than SNPs. Even with the usual methods of error reduction and detection in microsatellite genotyping there is still potential for map inflation and incorrect marker orders. The results of this study emphasise the importance of careful examination of CHROMPIC outputs to identify possible genotype errors, which can create spurious double recombinants. We also suggest that the least error prone markers (SNPs) should be mapped before adding the microsatellites, at least until it is computationally practical to FLIP greater than 5 markers at a time.

This study has highlighted the importance of knowing how much effect error rates have had on the final outcome of a linkage map. With map inflation likely to be present in other microsatellite-based studies, it remains to be seen whether subsequent SNP mapping projects will reveal previously undetected errors in map order or length. However, although microsatellites are more error prone than SNPs, their widespread availability, ease of typing, high polymorphism and their cross-species utility will ensure that they remain useful in linkage-mapping studies, especially for comparing the genomes of non-model organisms. In summary, we are not suggesting that microsatellites should be abandoned in mapping studies, but we do urge that appropriate error checking precautions are employed.

## Authors' contributions

AB participated in the microsatellite discovery, carried out the microsatellite genotyping and linkage map construction and drafted the manuscript. JStapley participated in the SNP linkage map construction, provided the SNP genotyping and co-drafted the manuscript. DAD participated in the microsatellite discovery and primer design. TRB developed and managed the zebra finch mapping pedigree. TB participated in the conception and design of the study. JSlate conceived of the study and participated in its design and coordination, as well as co-drafting the manuscript. All authors read and approved the final manuscript.

## Supplementary Material

Additional file 1 **Table S1. Characterisation of microsatellite markers. **Characterisation of 26 zebra finch (*Taeniopygia guttata*) microsatellite markers used to create the linkage maps for the zebra finch chromosomes 1, 1A, 2, and 9.
T_m_, melting temperature (calculated using PRIMER3)
EST, expressed sequence tag
†, all primer sequences were identical to zebra finch sequence (obtained either via trace files or assembled genomic sequences)
a, Excluded after CHROMPIC revealed an excess of double recombination events with adjacent markers, indicative of high error rate.
b, Excluded from linkage maps as parent-offspring mismatches estimated the error rate > 0.02
c, Could not be accurately scored after multiplexing Click here for file

Additional file 2 **Table S2. Results of linkage mapping. **Summary of the linkage map results for four zebra finch *(Taeniopygia guttata) *chromosomes (Tgu1, Tgu1A, Tgu2 and Tgu9). The predicted positions of all markers on the zebra finch genome sequence after a BLAST analysis are included. Linkage map positions highlighted in bold are the framework loci that are supported at a LOD score ≥ 3 for that linkage map. Click here for file
